# Cost of palliative radiation to the bone for patients with bone metastases secondary to breast or prostate cancer

**DOI:** 10.1186/1748-717X-7-168

**Published:** 2012-10-12

**Authors:** Gregory Hess, Arie Barlev, Karen Chung, Jerrold W Hill, Eileen Fonseca

**Affiliations:** 1 IMS, One IMS Drive, Plymouth Meeting, PA, 19462, USA; 2 Leonard Davis Institute, University of Pennsylvania, Colonial Penn Center, 3641 Locust Walk, Philadelphia, PA, 19104, USA; 3 Amgen Inc, One Amgen Center Drive, Thousand Oaks, CA, 91320, USA

**Keywords:** Cost, Palliative radiation, Bone metastases secondary to breast or prostate cancer

## Abstract

**Background:**

To estimate the costs (paid amounts) of palliative radiation episodes of care (REOCs) to the bone for patients with bone metastases secondary to breast or prostate cancer.

**Methods:**

Claims-linked medical records from patients at 98 cancer treatment centers in 16 US states were analyzed. Inclusion criteria included a primary neoplasm of breast or prostate cancer with a secondary neoplasm of bone metastases; ≥2 visits to ≥1 radiation center during the study period (1 July 2008 through 31 December 2009) on or after the metastatic cancer diagnosis date; radiation therapy to ≥1 bone site; and ≥1 complete REOC as evidenced by a >30-day gap pre- and post-radiation therapy.

**Results:**

The total number of REOCs was 220 for 207 breast cancer patients and 233 for 213 prostate cancer patients. In the main analysis (which excluded records with unpopulated costs) the median number of fractions per a REOC for treatment of metastases was 10. Mean total radiation costs (i.e., radiation direct cost + cost of radiation-related procedures and visits) per REOC were $7457 for patients with breast cancer and $7553 for patients with prostate cancer. Results were consistent in sensitivity analyses excluding patients with unpopulated costs.

**Conclusions:**

In the US, current use of radiation therapy for bone metastases is relatively costly and the use of multi-fraction schedules remains prevalent.

## Background

Bone metastasis, the most common cause of cancer-related pain, [1-3] occurs in up to 90% of patients with advanced breast or prostate cancer [1,2,4,5]. Median survival times for patients with breast and prostate cancer after diagnosis of bone metastasis range from 24 to 36 months [6-8]. Care of bone metastases is directed at decreasing skeletal complications, delaying or relieving pain, avoiding toxicity, and maintaining functional independence [2,9,10]. Treatment for the care of bone metastases can include surgery, radiotherapy, analgesics, anti-resorptive agents (e.g., denosumab, bisphosphonates), and radiopharmaceuticals. The prevailing treatment modality for bone metastases is radiation therapy, which is recommended by the National Comprehensive Cancer Network (NCCN) clinical oncology practice guidelines for use after or in conjunction with denosumab or bisphosphonates in treatment of patients with breast or prostate cancer [11,12]. Radiation therapy has been demonstrated to be effective in pain relief and in reducing pathological fractures and spinal cord compression, which may lead to functional improvements [2,13].

Radiation treatment to bone in patients with bone metastases contributes significantly to the overall burden of cancer therapy [14,15] and the socioeconomic impact is expected to increase with the aging of the populations of developed countries [16]. Of note, approximately half of the Medicare costs for patients with cancer are incurred during the last 60 days of life [15]. While it is one of the most common treatment modalities, relatively little is known about the economic burden of palliative radiation therapy for bone metastases. Certain economic modeling studies suggest that single-fraction radiation therapy is more cost effective than pain medication, chemotherapy, and multifraction radiation therapy [17-20]. However, these studies primarily used data from clinical trials or the medical literature with estimated charges and costs based on the literature, expert opinion, or patient surveys; as estimates, their results may not reliably reflect the actual cost of radiation therapy as used in clinical practice. The intent of the study reported herein was to estimate and establish, based on current clinical practice data, the actual costs (paid amounts, rather than charged or billed) of palliative radiation episodes of care (REOCs) for bone metastases secondary to breast or prostate cancer. Total radiation costs, radiation direct costs, and the costs of radiation-related visits and procedures associated with the REOC are reported.

## Materials and methods

### Design and setting

This retrospective, observational study was conducted utilizing data available from a total of 98 radiation treatment centers in 16 states in the United States; 96% freestanding and 4% hospital-based. These centers provide a range of radiation therapy services and also provide global billing, which includes professional and technical charges and paid amounts within a single billing process. Data were extracted from claims-linked electronic medical records (EMR) from the radiation treatment centers. Each center utilizes the same EMR/billing software with a copy of the data maintained at a central data repository, which enabled the study. Data were de-identified and certified as being compliant with the Health Insurance Portability and Accountability Act (HIPAA).

### Sample

Records from patients with a primary neoplasm of breast or prostate cancer (identified by their respective International Classification of Diseases, 9^th^ revision [ICD-9] codes) and a secondary neoplasm of bone metastases (ICD-9, code 198.5) were analyzed. Selection criteria for qualifying patients included the following:

1. A tumor type of interest (breast or prostate)

2. Radiation center visit date between 7/1/2008 and 12/31/2009

3. Known age and gender

4. A secondary neoplasm of bone metastases

5. At least one cost (paid) record with a monetary value greater than zero dollars

6. ≥2 radiation visits on or after the bone metastases diagnosis date during the study period

7. Radiation therapy to ≥1 bone site (spine, hip, femur, skull, humerus, pelvis, shoulder, clavicle, or other specified osseous site)

8. Patients with ≥1 completed REOC as evidenced by a >30-day gap in radiation therapy

9. Patients with <16 fractions during any REOC (i.e. exclusion of regimens that likely reflected miscoding and/or radiation to the soft tissues)

**Figure 1:**
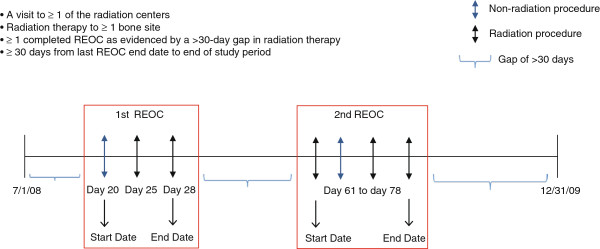
**Determining a Radiation Episode of Care (REOC).**

A REOC was defined as the first recorded visit to the radiation center, the index date, through the last visit to the center for that episode; the last visit was identified by a gap of >30 days for any visit or procedure, or recorded death (Figure 1). The 30 day duration of a gap was chosen based on analyses of the data showing that >96% of the qualifying study patients had less than a 30-day gap between visits.

### Statistical analysis

The REOC was the unit of analysis for the outcomes of interest. Patients could be followed across multiple qualifying REOCs and could contribute ≥1 REOC to the analysis. Outcomes of interest included radiation therapy exposure, healthcare resources utilized, and the associated *paid* amounts. Radiation therapy exposure was quantified as the total dose (Gy) (i.e., the sum of all doses received during a REOC); mean dose was assessed by treatment, by patient, and by fraction (defined as a unique radiation treatment); total fractions; mean and median fractions; frequency of treatments (i.e., more often than daily, less often than daily, or daily); count of treatments by site radiated; and REOC duration. Healthcare resources utilized during the REOC were assessed as visits (including consultation, simulation, treatment planning, and treatment), procedures, and all other healthcare resources associated with radiation therapy for metastatic bone lesions. A visit was defined as a unique treatment or consultation. Patients could have ≥1 visit in a day. The associated paid amounts were the monetary payments recorded from the patient and/or a third-party payer in the qualifying records.

Radiation therapy exposure characteristics, healthcare resources utilized, and paid amounts as well as demographics and clinical characteristics of the sample were summarized with descriptive statistics by tumor type (i.e., metastatic breast or prostate cancer). For analyses of costs, records were excluded if they had any unpopulated or absent costs in the main data analysis which most likely represented missing values or capitated amounts. To estimate the confidence interval for the means, a bootstrap analysis of costs utilizing 1000 replications was performed. Furthermore, to determine how unpopulated payment records could affect the results, a sensitivity analysis was conducted in which patients with any unpopulated or absent costs were excluded.

## Results

### Demographics

Ninety-six percent of patients with breast or prostate cancer in the study were treated at a free standing site and four percent at a hospital-based site.

The number of patients in the main analysis was 207 for the breast cancer stratum and 213 for the prostate cancer stratum. Table 1 shows demographics by cancer stratum for the main analysis. Overall, the majority of patients were ≥65 years old and had Medicare and/or commercial insurance.

**Table 1:** Baseline clinical characteristics and radiation exposure (main analysis)

	**Breast cancer**	**Prostate cancer**
Patients with radiation to the bone, n	207	213
**Demographics**		
Mean age, years (SD)	65 (12.5)	74 (8.4)
Age, n (%)		
<65 years	93 (44.9)	31 (14.6)
≥65 years	114 (55.1)	182 (85.4)
Female, n (%)	202 (97.6)	n/a
Payor,* n (%)		
Medicare	103 (49.8)	133 (62.4)
Commercial	94 (45.4)	64 (30.0)
Medicaid	7 (3.4)	3 (1.4)
Other	3 (1.4)	13 (6.1)
**Radiation profile**		
Total REOCs, n	220	233
Length of treatment, days		
Mean (SD)	30 (16.0)	30 (16.7)
Median	26	26
Dose, cGy		
Mean by episode	3207	3301
Mean per patient	3409	3611
Mean by fraction	327	337
Radiation schedule by REOC, n		
Daily	201	203
Greater than daily	7	13
Less than daily**	0	0
Site radiated by REOC*** n (%)		
Femur	14 (6.8)	28 (13.1)
Hip	45 (21.7)	40 (18.8)
Humerus	3 (1.4)	9 (4.2)
Pelvis	20 (9.7)	34 (16.0)
Rib	12 (5.8)	12 (5.6)
Sacrum	21 (10.1)	17 (8.0)
Skull	9 (4.3)	10 (4.7)
Spine	97 (46.9)	95 (44.6)
Other bone sites	28 (13.5)	27 (12.7)
Total fractions	2173	2344
Fractions by REOC		
Mean (SD)	9 (5)	10 (4)
Median	10	10
Fractionation schedule,^†^ n		
1	9	10
4 to 5	21	23
10	35	43
15	47	47
**Other**	**102**	**96**
Fractions by site,^‡^ n		
Femur	10.2	9.7
Hip	10.0	9.6
Humerus	9.0	10.3
Pelvis	8.7	8.1
Rib	9.4	5.0
Sacrum	10.6	10.9
Skull	11.3	10.5
Spine	9.9	10.2
Other bone site	8.8	10.4

*A patient could have multiple payor coverage for the same procedure.

** Every other day.

*** A patient could be counted multiple times with radiation to the same site or to multiple sites.

^†^ Fractionation schedule is the count of unique patients by the range of total number of fractions across REOCs.

^‡^ Fractions by site are a sum of all fractions received across REOCs stratified by bone site.

### Radiation therapy exposure

Table 1 contains the summary statistics of palliative radiation therapy exposure for the treatment of metastatic bone lesions in the main analysis. The total number of REOCs was 220 for the 207 breast cancer patients and 233 for the 213 prostate cancer patients. The mean duration of radiation therapy during a REOC was 30 days. Independent of cancer type, the majority of patients received at least 10 fractions across REOCs. The most common site radiated was the spine, which received the greatest number of fractions across REOCs for both breast and prostate cancer.

### Healthcare resource utilization and costs

Table 2 shows summary statistics on healthcare resource utilization in the main analysis. The mean number of visits by REOC was 13, which was the same for both breast and prostate cancer patients. For breast and prostate cancer respectively, 72.9% and 71.7% of REOC visits involved radiation treatment, with the remainder reflecting visits for radiation-related procedures. Radiation treatment was defined as the actual delivery of radiotherapy, as well as the most commonly observed, associated procedures on the same visit, patient record: medical radiation physics consultation, computed tomography guidance for placement, and radiation calculations. Radiation-related procedures were all other procedures including: treatment devices, design and construction, complex (CPT/HCPCS code 77334); therapeutic radiology simulation-aided field setting, 3-dimensional (CPT/HCPCS code 77295); therapeutic radiology simulation-aided field setting, simple (CPT/HCPCS code 77280); and special dosimetry, when prescribed by the treating physician (CPT/HCPCS code 77331).

**Table 2:** Healthcare resource utilization (Main Analysis)

**Patients**	**Breast cancer**	**Prostate cancer**
	**n=207**	**n=213**
**Visits**		
Visits by REOC*		
Mean (SD) number of visits	13 (5.8)	13 (5.8)
Median number of visits	13	14
% visits with radiation	72.9	71.7
**Procedures**		
Bone sites radiated by REOC		
Patient count	207	213
Total	4607	4739
Mean (SD)	20.9 (12)	20.3 (12)
Median (range)	19 (1–49)	17 (1–58)
Radiation-related procedures†		
Patient count	203	212
Total procedures	1843	1984
Mean (SD) procedures	8.4 (3)	8.5 (4)
Median (range) procedures	8 (1–23)	8 (1–32)
Radiation+radiation-related procedures		
Patient count	207	213
Total procedures	6450	6723
Mean (SD) procedures	29.3 (13)	28.9 (13)
Median (range) procedures	28 (1–66)	26 (1–76)

* A visit was a unique treatment or consultation. A patient could have multiple visits in a day.

Total resources used from the time of initial bone treatment through the end of the study period.

† Radiation treatment included the actual delivery of radiotherapy, as well as the most commonly observed, associated procedures on the same visit, patient record: medical radiation physics consultation, computed tomography guidance for placement, and radiation calculations. Radiation-related procedures were all other procedures including: treatment devices, design and construction, complex (CPT/HCPCS code 77334); therapeutic radiology simulation-aided field setting, 3-dimensional (CPT/HCPCS code 77295); therapeutic radiology simulation-aided field setting, simple (CPT/HCPCS code 77280); and special dosimetry, when prescribed by the treating physician (CPT/HCPCS code 77331).

**Figure 2:**
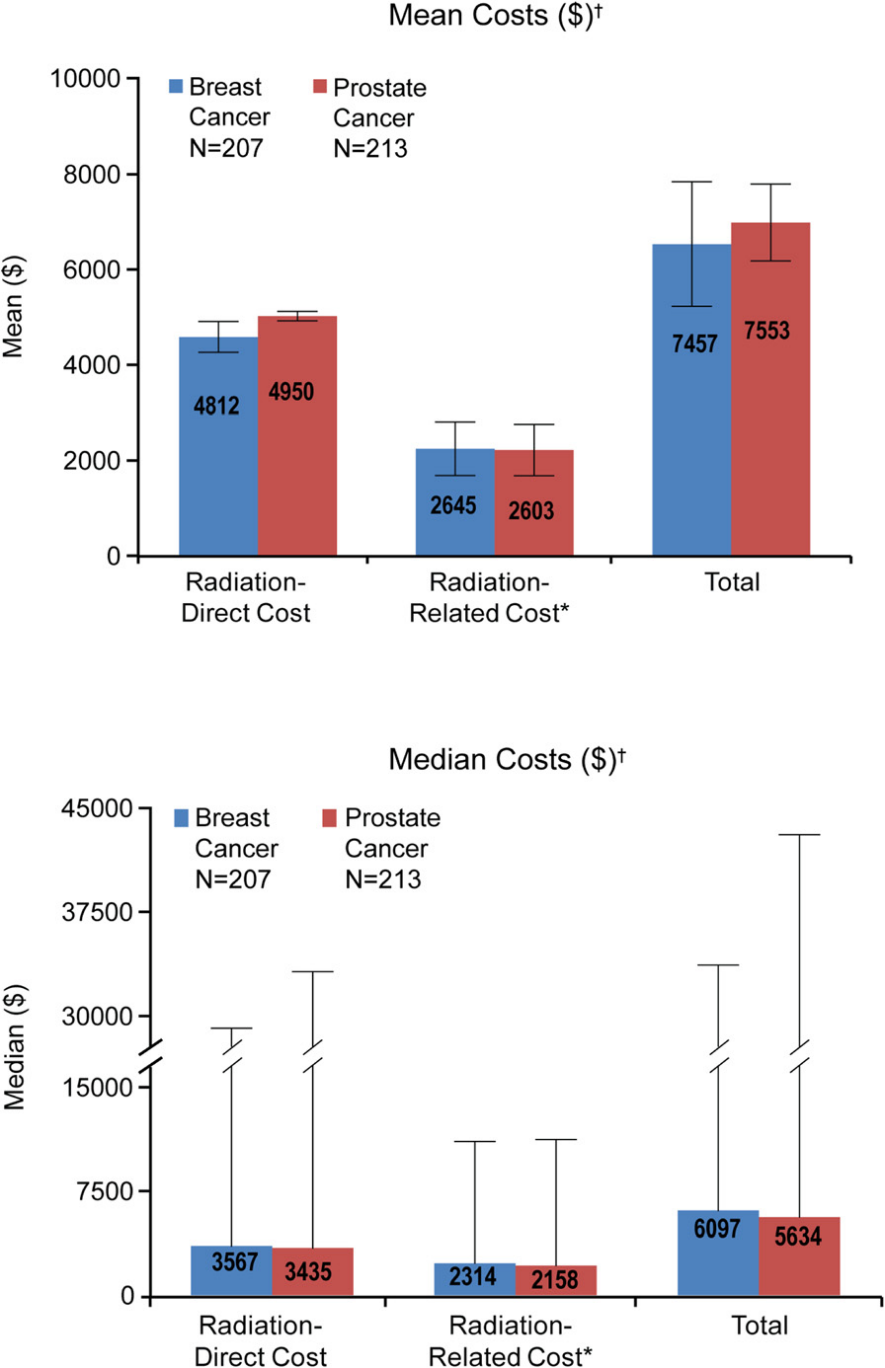
**Main analysis of healthcare costs ($) per REOC.** *Radiation-related procedures were visits and procedures at radiation center excluding actual administration of radiation. Radiation-related procedures included intensity-modulated treatment delivery, single or multiple fields/arcs, via narrow spatially and temporally modulated beams, binary, dynamic multileaf collimator, per treatment session (Current Procedural Terminology/Healthcare Common Procedure Coding System [CPT/HCPCS] code 77418); treatment devices, design and construction, complex (CPT/HCPCS code 77334); therapeutic radiology simulation-aided field setting, 3-dimensional (CPT/HCPCS code 77295); therapeutic radiology simulation-aided field setting, simple (CPT/HCPCS code 77280); special dosimetry, only when prescribed by the treating physician (CPT/HCPCS code 77331). ^†^Excluded records with unpopulated costs.

Figure 2 shows summary statistics on healthcare costs for the main or primary analysis (which excluded records with unpopulated costs i.e. absent value, null or zero) and Table 3 shows the results for the bootstrap analysis of these costs. The numbers of patients included in the main analysis were 207 for breast cancer and 213 for prostate cancer. The numbers of patients included in the sensitivity analysis were 91 for breast cancer and 89 for prostate cancer. In the main analysis, mean total radiation costs per REOC (i.e., direct radiation cost + cost of radiation-related procedures and visits) were $7457 for breast cancer patients and $7553 for prostate cancer patients. The corresponding numbers for the sensitivity analysis were $7457 for breast cancer and $6936 for prostate cancer. Mean and median total radiation costs per REOC were generally similar between breast cancer patients and prostate cancer patients (Figure 2). Within the main analysis, the median costs were $6097 for patients with breast cancer and $5634 for patients with prostate cancer. While in the sensitivity analysis, median costs were $6332 for patients with breast cancer and $5655 for patients with prostate cancer. Overall, the results did not materially differ between the main analysis and the sensitivity analysis.

**Table 3:** Bootstrap estimates of standard errors (SEs) and 95% confidence intervals for costs ($)

	**Observed**	**Bootstrap**	**95% Confidence interval**	
	**mean, $**	**SE**	**Lower**	**Upper**
**Breast cancer**				
Radiation direct costs	4812	298	4229	5395
Radiation-related costs*	2645	124	2402	2889
***Total radiation costs***	***7457***	***383***	***6707***	***8208***
**Prostate cancer**				
Radiation direct costs	4950	325	4314	5587
Radiation-related costs*	2603	118	2372	2834
***Total radiation costs***	***7553***	***410***	***6749***	***8357***

* Visits and procedures at radiation center excluding actual administration of radiation.

## Discussion

Increasing limitations on healthcare resources and the recognition that treatment of bone metastases contributes significantly to the socioeconomic burden of cancer have led to calls to evaluate and better understand costs associated with treatment [14-16]. This study quantified the recorded, actual, and paid costs of palliative radiation therapy for bone metastases secondary to breast or prostate cancer. Results from the analysis demonstrate that the total cost of radiation therapy for bone metastases was substantial. Mean total radiation costs (i.e., direct radiation cost + cost of radiation-related procedures and visits excluding actual radiation) per REOC were $7457 for breast cancer patients and $7553 for prostate cancer patients. Radiation direct costs accounted for the majority of total radiation costs. Total radiation costs did not differ materially between breast cancer and prostate cancer. Likewise, total radiation costs did not differ materially as a function of how unpopulated payment records were accounted for (i.e., records with unpopulated costs excluded from the main analysis; patients with unpopulated cost records excluded from the sensitivity analysis).

Palliative radiation practices including length of treatment, dose, radiation schedule, fractions by REOC, and frequency of fractions, appear to be similar for the treatment of patients with breast or prostate cancer. Likewise, the bone sites radiated during a REOC were also similar for the treatment of patients with breast or prostate cancer. The most commonly irradiated bone sites for both cancer types were the spine and the hip, and the least common was the humerus; most likely reflecting the variations in incidence, surrounding normal-tissue tolerance, extent of metastatic lesion(s), and other factors.

In addition to quantifying the costs of radiation therapy as used in current clinical practice, the results of this study sheds light on US practice patterns of radiation to bone in patients with bone metastases. Current guidelines indicate that radiotherapy is a successful and efficient method by which to palliate pain and/or prevent the morbidity of bone metastases [21]. For the majority of patients with breast or prostate cancer, the median number of fractions per REOC was 10; a result reflecting common use of multifraction therapy. Clinical evidence, including that from three large, randomized trials of single-fraction therapy (8 Gy) vs. multifraction therapy (20 to 30 Gy), demonstrates that single-fraction therapy may be as effective as multifraction therapy for the treatment of bone metastases [21-24]. Although single-fraction therapy may be as clinically effective, more convenient for the patient, and less expensive than multifraction therapy, single-fraction therapy has not currently been accepted as standard practice in the US [25,26]. A contributing factor may be the durability of the pain response as multifraction regimens (10 fractions vs. 1 fraction) have been associated with less re-treatment. This point is supported by the median and mean numbers of fractions observed per REOC for patients with breast or prostate cancer in the current study, which indicates that the use of multi-fraction schedules in the management of patients with advanced breast or prostate cancer and bone metastases is common, and is likely associated with a substantial patient burden.

Strengths of this study include its geographical diversity (data from 98 radiation cancer treatment centers in 16 US states), reflection of recent practice patterns (study period from 1 July 2008 to 31 December 2009), and “real-world” reflections (data based on electronic medical records from freestanding and hospital-based cancer treatment centers). However, the results reflect centers in 16 states and it is unknown if other centers, particularly in the other 34 states, would present similar findings. Limitations, typical of retrospective, claims-based studies, include the potential for coding errors and underreported or missing information on historical radiation treatment. In addition, the limitation of incomplete patient records, a typical shortcoming of claims-based studies, was addressed in the current study by undertaking a sensitivity analysis where patients were excluded if they had any unpopulated or absent cost records. The results on healthcare resource use and the cost data were similar and followed a consistent pattern across analysis methods, suggesting that the occurrence of incomplete payment records did not systematically affect the results. The results are specific to patients with bone metastases treated with radiation therapy, and the cost for those not treated with radiation was not studied. Lastly, it is important to note that these results describe an ‘average’ patient, and we know as clinicians and researchers that patients are individuals that follow a broad range of patterns.

Future research to examine concurrence or variations of these findings within other states will be beneficial, as well as exploring additional areas such as the correlation of the number of radiation procedures by site irradiated with increased cost, the relative costs of various radiation regimens and fractionation schedules, the cost-effectiveness of radiation therapy compared to alternative therapies for bone metastases, costs incurred without radiation therapy e.g. cord compression, and cost differences between private payers, Medicare and Medicaid, and/or academic and non-academic facilities.

## Conclusions

The results of this study show that in the United States, palliative radiation treatment of bone metastases in patients with breast or prostate cancer represents a significant cost burden. Furthermore, the study provides a foundation for evaluating the relative costs of current and investigational therapies for the treatment of bone metastases.

## Competing interests

Gregory Hess, Jerrold Hill, and Eileen Fonseca were employees of SDI when this study was conducted. SDI received research funding from Amgen Inc.

Arie Barlev and Karen Chung are employees of Amgen Inc. and own Amgen stocks.

## Authors' contributions

GH, AB, and KC were responsible for the conception of the manuscript. GH, JH, and EF made central contributions to the study design, data acquisition, and analysis. GH, AB, KC, JH, and EF substantially contributed to the developing, drafting, editing, and revising of the manuscript. All authors have read and approved of the final manuscript.
